# Evaluation of Atrial Fibrillation Detection in Short-Term Photoplethysmography (PPG) Signals Using Artificial Intelligence

**DOI:** 10.7759/cureus.45111

**Published:** 2023-09-12

**Authors:** Debjyoti Talukdar, Luis Felipe De Deus, Nikhil Sehgal

**Affiliations:** 1 Medical Research, Mkhitar Gosh Armenian-Russian International University, Yerevan, ARM; 2 AI Research, Vastmindz Limited, London, GBR

**Keywords:** persistent atrial fibrilation, paroxysmal atrial fibrillation, heart rate variability (hrv), irregular heartbeats, arrhythmia, artificial intelligence, signal processing, ecg signals, photoplethysmography, atrial fibrillation

## Abstract

Background

Atrial fibrillation (AFIB) is a common atrial arrhythmia that affects millions of people worldwide. However, most of the time, AFIB is paroxysmal and can pass unnoticed in medical exams; therefore, regular screening is required. This paper proposes machine learning (ML) methods to detect AFIB from short-term electrocardiogram (ECG) and photoplethysmography (PPG) signals.

Aim

Several experiments were conducted across five different databases, with three of them containing ECG signals and the other two consisting of only PPG signals. Experiments were conducted to investigate the hypothesis that an ML model trained to predict AFIB from ECG segments could be used to predict AFIB from PPG segments.

Materials and methods

A random forest (RF) ML algorithm achieved the best accuracy and achieved a 90% accuracy rate on the University of Mississippi Medical Center (UMMC) dataset (216 samples) and a 97% accuracy rate on the Medical Information Mart for Intensive Care (MIMIC)-III datasets (2,134 samples).

Results

A total of 269,842 signal segments were analyzed across all datasets (212,266 were of normal sinus rhythm (NSR) and 57,576 corresponded to AFIB segments).

Conclusions

The ability to detect AFIB with significant accuracy using ML algorithms from PPG signals, which can be acquired via non-invasive contact or contactless, is a promising step forward toward the goal of achieving large-scale screening for AFIB.

## Introduction

Atrial fibrillation (AFIB) is an arrhythmia characterized by the absence of the P wave (which represents the electrical depolarization of the atria) and irregular heartbeats. AFIB is also the most common sustained heart rhythm disorder [1]. Commonly, it is associated with deadly and debilitating consequences, including heart failure, stroke, poor mental health, reduced quality of life, and death [2]. According to a systematic review published in The Lancet journal, the prevalence of AFIB is estimated to be 46.3 million globally, with 3.8 million new diagnoses annually; this represents an increase of 32% from 2006 to 2016 [3]. Moreover, it has been estimated that six to 12 million people will suffer from this condition in the US by 2050 and 17.9 million in Europe by 2060 [4].

There are four different types of AFIB conditions: paroxysmal AFIB, persistent AFIB, long-standing persistent AFIB, and permanent AFIB. However, as stated by the National Heart, Lung, and Blood Institute [5], only the severest, long-standing persistent, and permanent can be easily detected with an electrocardiogram (ECG) exam, while the others are harder to identify due to the irregularity of the symptoms.

These irregularities make the diagnosis of AFIB very difficult; therefore, models that can predict AFIB events with short ECG signals have been developed. Those models are usually based on digital signal processing and artificial intelligence (AI) techniques. The main goal of these approaches is to make an embedded solution that could be used in mobile phones, smartwatches, or smart bands.

Researchers have put their efforts into two main branches of the AI field: machine learning (ML) and deep learning. In the past few years, deep learning has become a hot topic in the context of computing and is widely applied in various application areas such as healthcare and visual recognition [6]. Convolution neural networks (CNNs) have been used to detect AFIB [7]; the method proposed by Ross-Howe et al. uses ECG signals from the Massachusetts Institute of Technology & Beth Israel Hospital (MIT-BIH) Atrial Fibrillation Database v1.0 [8]. Spectrograms were computed from a 6-s ECG window, and the CNN was then trained using these spectrograms. This method achieved a reported sensitivity of 98.33%, specificity of 89.74%, and accuracy of 93.16%.

Bruun et al. [9] have used ML models combining heart rate variability (HRV) and discrete wavelet transform features. ECG signals from the MIT-BIH dataset were segmented using a 30-s sliding window with a 5-s stride. Features were extracted from each window and used to create a bootstrap ensemble method with aggregation on decision trees. This method achieved a reported sensitivity of 87.97%, specificity of 96.62%, and accuracy of 93.33%.

There are other authors who have tried to combine the advantages of deep neural networks with handcrafted features. Hu et al. [10] have used the Atrial Fibrillation Classification from a Short Single Lead ECG Recording: The PhysioNet/Computing in Cardiology Challenge 2017 [11] dataset to extract multiple features. The handcrafted features represent HRV, morphological, frequency domain, and others. A residual neural network (ResNet) was used to extract deep features from a single heartbeat, and after training the ResNet, the in-depth features of the last layer were extracted. Ultimately, a random forest model with 1,000 classifiers was trained using the handcrafted and deep features. Reported results show a sensitivity of 88.70%, specificity of 99.60%, and accuracy of 96.30%.

The aim of this study is to develop and compare two different techniques to detect AFIB events in short-term ECG signals, as well as verify the feasibility of using PPG signals. The primary method is based only on signal processing, while the secondary method uses AI models to predict AFIB.

This article was previously posted to the medRxiv preprint server on March 8, 2023.

## Materials and methods

Datasets

In this work, five different datasets were used, and all of them are publicly available. The datasets used are from Physionet’s database [12], named MIT-BIH Atrial Fibrillation Database v1.0 (AFDB) [8], AFIB Classification from a Short Single Lead ECG Recording: The PhysioNet/Computing in Cardiology Challenge 2017 (AFC) [11], MIT-BIH Normal Sinus Rhythm Database (NSRDB), MIMIC-III Dataset [13], and UMMC (UMass Memorial Medical Center) Simband Dataset [14]. Each one of them will be described in the following sections.

AFDB

This dataset consists of 23 long-term ECG recordings, and each sample has around 10 hours of ECG signals sampled at 250 Hz, with 12-bit resolution over a range of ±10 mV. Alongside the ECG signal, there are annotation files that were manually prepared by physicians that contain rhythm annotations of types AFIB, AFL (atrial flutter), J (AV junctional rhythm), and N (normal sinus rhythm).

In this work, only normal and AFIB events were used. Since the dataset has long-term signals with multiple normal and AFIB events, using the annotation file provided, the signals were divided into events and treated individually. For example, if in a given signal there is normal sinus rhythm from sample 0 to 10,000 and then AFIB from 10,001 to 20,000, this signal will be divided into two blocks and treated as individual samples.

AFC

The 2017 PhysioNet/CinC Challenge dataset was created to encourage the development of algorithms to classify, from a single short-term ECG lead recording, whether the recording shows normal sinus rhythm, AFIB, an alternative rhythm, or is too noisy to be classified. The available training set contains 8,528 single lead ECG recordings lasting from 9 s to just over 60 s, sampled at 300 Hz. In this work, only signals classified as normal or AFIB were used.

NSRDB

This database includes 18 long-term ECG recordings of subjects that have had no indication of significant arrhythmia. Data include five men, aged 26-45, and 13 women, aged 20-50. Each sample has about 25 hours of ECG signals sampled at 128 Hz. However, because of the large amount of data, only two hours of data were used.

MIMIC-III Dataset

The Medical Information Mart for Intensive Care (MIMIC)-III is a large, single-center database comprising information relating to patients admitted to critical care units at a large tertiary care hospital. This database provides continuous ECG and photoplethysmography (PPG) signals from patients in critical care at a large tertiary care hospital [13]. Ten subjects were randomly selected from the MIMIC-III database by Han et al. [14] and segmented in windows of 30 s long. In this study, only PPG signals were used, which were downsampled to 50 Hz.

UMMC Simband Dataset

The UMMC dataset [14] consists of 37 patients (28 male and nine female) with cardiac arrhythmia, aged between 50 and 91 years old. The participants were asked to wear the smartwatch Simband 2 (Samsung Digital Health, San Jose, CA, USA) and had their ECG reference taken using a seven-lead Holter monitor (Rozinn RZ153+ Series; Rozinn Electronics Inc., Glendale, NY, USA). Data were preprocessed and segmented in 30-s windows with no overlapping; ECG data were sampled at 128 Hz, while PPG data were downsampled to 50 Hz. All the signals were labeled within five categories: 0-normal sinus rhythm (NSR), 1-AFIB, 2-premature atrial contractions/premature ventricular contractions (PAC/PVC), 3-not sure if it is NSR or PAC/PVC, 4-noisy PPG, and not a number (NaN)-No nCG reference. However, only PPG signals were used in this study, separated into two labels, AFIB (1) and normal (0,2,3); labels 4 and NaN were not used.

Preprocessing

Firstly, the raw ECG signal was obtained from the datasets. The raw ECG was filtered using a smoothing filter Savitzky Golay [15] of fourth order and 19 frames. The smoothed ECG signal was then filtered using a band-pass filter, a second-order Butterworth filter [16] with cutoff frequencies of 0.5 Hz-20 Hz. Ultimately, the signal was normalized using the MinMax approach, as described in Eq. 1.

$n[i]=10 \frac{s f[i]&minus;min(s f)}{max(s f) &minus; min(s f)}$ - (1)

• s n: Signal Normalized

• s f: Signal Filtered

Once the ECG signals were processed, the peaks of the R-wave were extracted. A custom peak detector algorithm was created alongside a double peak correction routine to cope with erroneous peaks.

**Figure 1:**
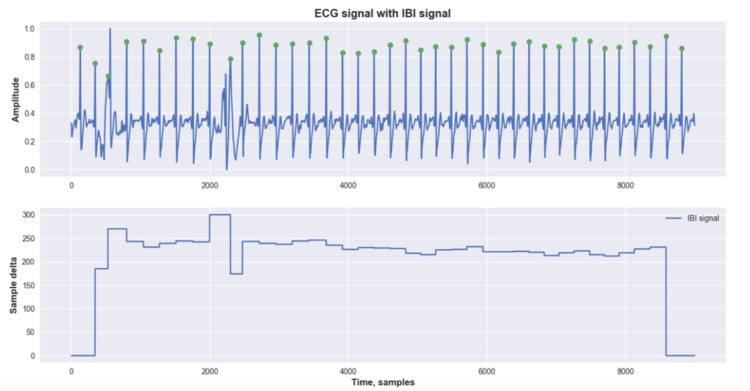
Example of preprocessing layer - electrocardiogram (ECG) signal with highlighted peaks and inter-beat-interval (IBI) signal Electrocardiogram (ECG), Inter-Beat-Interval (IBI)

The third step was to create the Inter-beat signal, which describes, in terms of samples, the distance in time between each peak. The signal, as shown in Figure 1, is normalized to the same length as the ECG signal, so each ECG sample has the correspondent inter-beat-interval (IBI).

The IBI signal carries information about changes in the cardiac rhythm. However, it is well-known that signals from public databases are not always perfect and may be noisy, which can lead to wrong predictions. Thus, we propose another routine to validate or invalidate parts of the signal based on the IBI signal. This routine works as a mask; where there are validated IBI segments, we kept the IBI; however, where there are in-validated IBI segments, we pruned the interval. Eq. 2 describes the conditions outlined to invalidate or keep corresponding IBI intervals.

$BI(t) = \{ibi, 0 \frac{max ibi \geq ibi \geq min ibi}{otherwise}$ - (2)

Once we obtained the validated IBI signal, a sliding window approach was used to search for reliable frames within the IBI signal. The frame is considered reliable if, in a given window size, there were not any invalidated segments, which means that those frames have good signal quality.

Digital signal processing method

The first method uses only signal processing techniques to identify AFIB events. In fact, most of the method’s functions were already described in section II B. After analyzing the IBI signal, as stated previously, frames of the ECG signal were extracted with an 8-s sliding window, along with a stride of 2 s. The method will analyze frame by frame and extract a new IBI signal for each one. The first derivative of each frame’s IBI signal was computed. The first derivative shows significant spikes where there were sudden changes in the IBI signal; thus, using a simple threshold, it is possible to identify abnormal changes in the cardiac rhythm.

For each frame, two parameters were extracted. The first was a Boolean that represents if this frame has at least one abnormal change and the second was a float number that represents the ratio of abnormal changes, which was calculated by using the ratio between abnormal spikes in the first derivative of the IBI signal and the number of peaks within this frame. Ultimately, this signal will be classified as AFIB if the mean ratio of abnormal changes between all frames is greater than a threshold.

ML method

Secondly, as stated previously, the most common approach to detect AFIB events in short-term signals is by using AI techniques. Therefore, this work proposes its own AI model to predict AFIB. The method uses the same preprocessing layer as stated previously in section II B. For each frame, instead of looking at the first derivative of the IBI signal, this method seeks to extract HRV features in a given window. This work has used a sliding window approach with 15 s in size and a 2-s stride.

The HRV features were used as an input to the ML models, and this work has evaluated the performance of three approaches: random forest (RF), support vector machine (SVM), and K-nearest neighbours (KNN). Seven different HRV features were selected based on permutation features importance:

• HRMAD: Mean absolute deviation of heart rate

• RMSSD: Root mean square of successive differences between normal heartbeats

• IRQNN: Inter-quartile range of normal heartbeats

• MCVNN: HRMAD divided by the median of normal heartbeats

• CVNN: Standard deviation of normal heartbeats divided by the mean of normal heartbeats

• CVSD: RMSSD divided by the mean of normal heartbeats

• HTI: HRV triangular index

The HRV features were extracted per frame and each entry represents one row with seven features and the corresponding label, AFIB or normal. However, given the sliding window approach, we may end up with multiple entries for the same individual, so it is essential to ensure the same individual is not included in both the training and test datasets.

**Figure 2:**
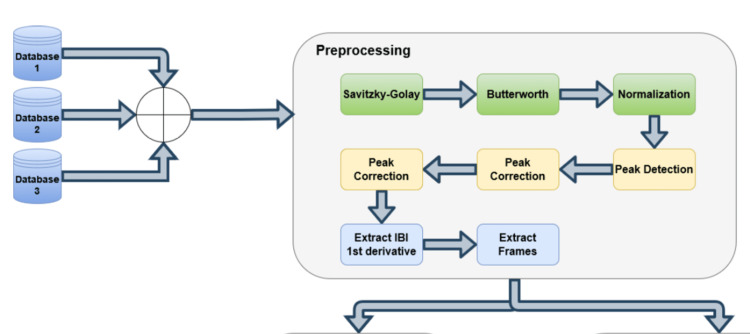
Block diagram, part 1 Interbeat Interval (IBI)

**Figure 3:**
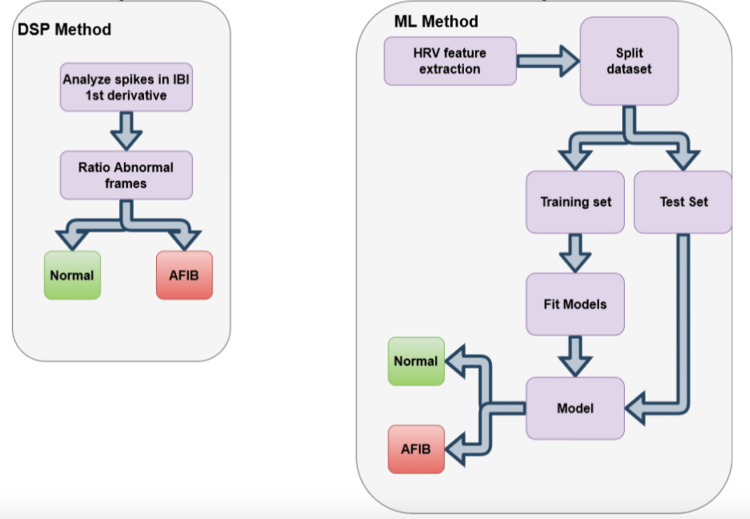
Block diagram, part 2 Interbeat Interval (IBI), Heart Rate Variability (HRV), Atrial Fibrillation (AFIB)

The dataset with HRV features was divided into two groups as follows: 60% of the individuals in the training set and 40% in the testing set. It is important to note that, when there is a varying number of entries for each individual, 60% of the individuals may not account for 60% of the total available data. Finally, Figures 2-3 show the respective block diagrams of the proposed approach. 

Metrics

In order to evaluate the performance of the proposed methods, we chose to use a set of objective metrics that will reflect the model’s performance. All the metrics, described in Eqs. 3, 4, 5, and 6, are based on the following concepts:

• True positive (TP): Event in which there was AFIB and it was correctly identified.

• False positive (FP): Event in which there was no AFIB and the algorithm pointed it incorrectly as AFIB.

• True negative (TN): Event in which there was no AFIB and the algorithm pointed it correctly as Non-AFIB.

• False negative (FN): Event in which there was AFIB and the algorithm has not found it.

$Accuracy = \frac{T P + T N}{TP +TN +FP +FN}$ - (3)

$Precision = \frac{TP}{TP + FP}$ - (4)

$Recall = \frac{TP}{TP + FN}$ - (5)

$F1Score = 2* \frac{Precision&lowast;Recall}{Precision + Recall}$ - (6)

## Results

**Figure 4:**
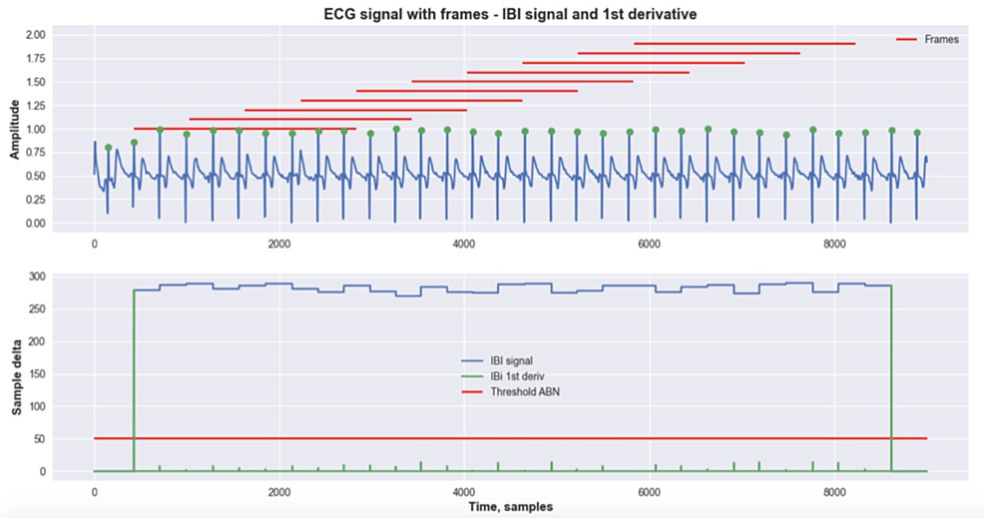
Example of the execution of the DSP method - normal ECG Digital Signal Processing (DSP), Electrocardiogram (ECG)

**Figure 5:**
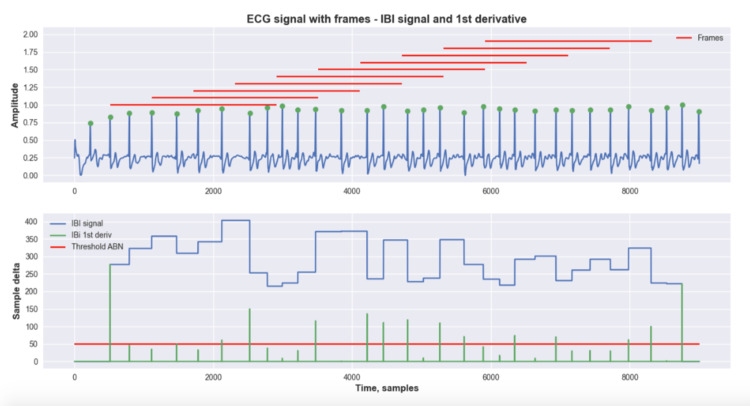
Example of the execution of the digital signal processing (DSP) method - atrial fibrillation (AFIB) electrocardiogram (ECG) Digital Signal Processing (DSP), Atrial Fibrillation (AFIB), Electrocardiogram (ECG)

Figures 4-5 show examples of the digital signal processing (DSP) method. In both figures, the top plot represents the ECG signal in blue with the highlighted peaks, and the red marks refer to the portion of the signal that was analyzed in each frame. In the bottom plot, the IBI signal is shown in blue, and its first derivative is shown in green; the red line represents the threshold for abnormal spikes. Figure 4 shows an example of a normal ECG, while Figure 5 shows an ECG with AFIB. One of the advantages of this method is that does not require any training process; thus, it is possible to use all the available data as a test set. Figure 5 shows an example of the execution of the DSP method - AFIB ECG. Furthermore, all three datasets were used individually as separate experiments. Figure 5 shows the class distribution for each dataset.

**Figure 6:**
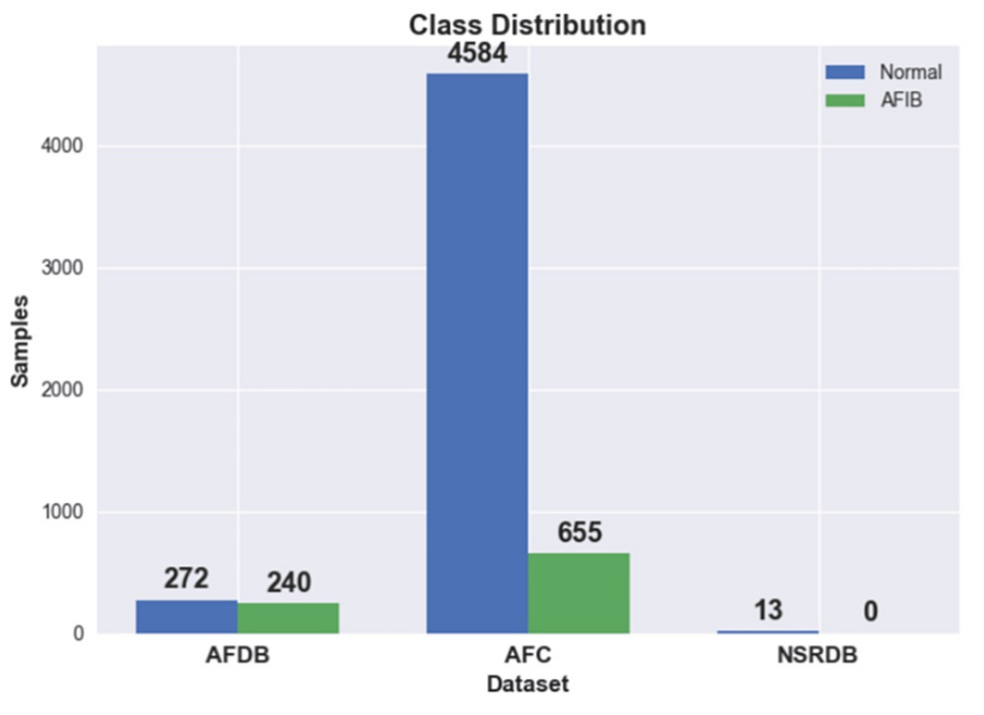
Class distribution in each dataset Normal Sinus Rhythm (NSR), Atrial Fibrillation (AF), Database (DB), Atrial Fibrillatory Contraction (AFC)

In the first experiment, data from the AFDB dataset were used. Originally, there were 23 long-term ECG samples; however, as stated previously, each signal was divided into blocks using the annotated events. Consequently, the number of samples increased to 512. Figure 6 shows the class distribution in each dataset.

**Figure 7:**
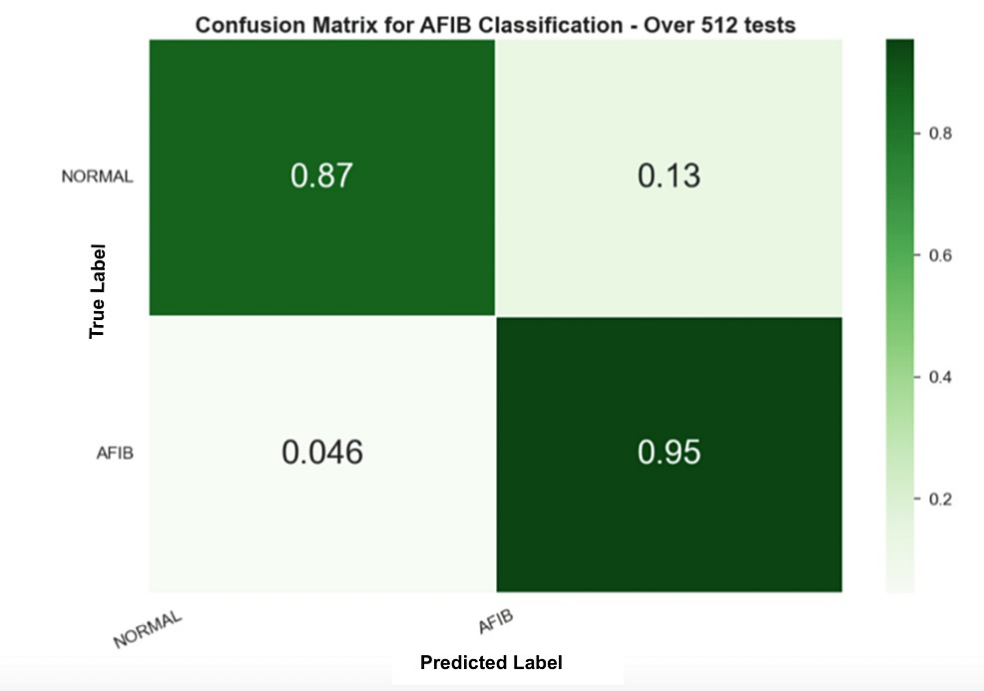
Confusion matrix for the DSP method applied to AFDB Digital Signal Processing (DSP), Atrial Fibrillation Database (AFDB)

Figure 7 shows the confusion matrix for the first experiment using the DSP method. In the first dataset, using the proposed metrics, the method was able to achieve 90.82% of accuracy, 90.98% of precision, 91.01% of recall, and an F1-score of 90.81%.

Secondly, using the AFC dataset, around 5,200 samples survived the preprocessing layer and were analyzed. Figure 7 shows the confusion matrix for the second experiment using the DSP method. In this experiment, the method was able to achieve 87.32% accuracy, 73.59% precision, 86.28% recall, and an F1-score of 77.49%.

Finally, in the last experiment of the first method, the NSRDB dataset was used to evaluate the false-positive rate. Thus, the purpose of this experiment was to see how many times the model would incorrectly classify segments as AFIB given there were only normal signals.

Since the normal sinus rhythm (NSRDB) database only has normal samples, neither a confusion matrix nor statistical results would be the best way to show the results, so we show the number of correct predictions, in 12 out of 13 samples, resulting in 92.31% accuracy.

**Figure 8:**
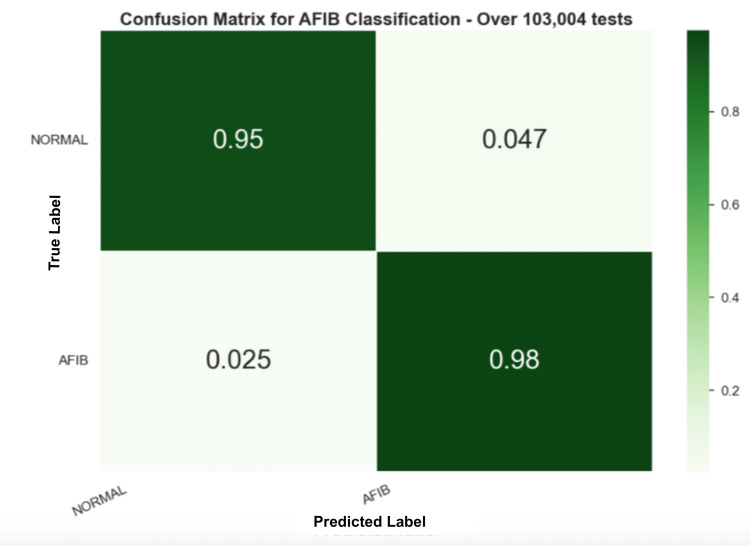
Confusion matrix for the ML method applied to the AFDB testing set Machine Learning (ML), Atrial Fibrillation Database (AFDB)

In the ML approach, HRV features were extracted from each frame of the signal. Hence, the resulting HRV data frame has multiple entries for one single sample, which means that the dataset class distribution also changes. Figure 8 shows the class distribution for the HRV datasets. Unlike the DSP method, ML does require a training process, which was carried out with 60% of the individuals from one of the datasets.

**Figure 9:**
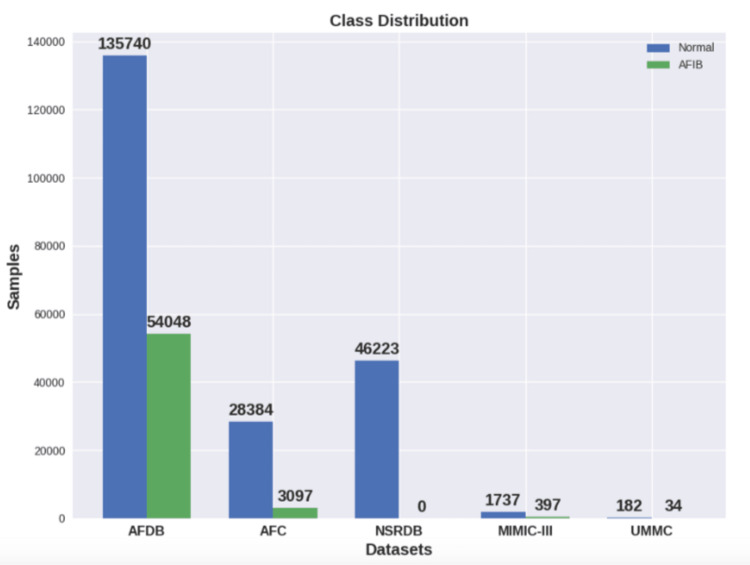
Class distribution in each dataset Atrial Fibrillation (AF) Database, Atrial Fibrillation Contraction (AFC), Normal Sinus Rhythm (NSR), University of Mississippi Medical Center (UMMC), Medical Information Mart for Intensive Care (MIMIC)

**Figure 10:**
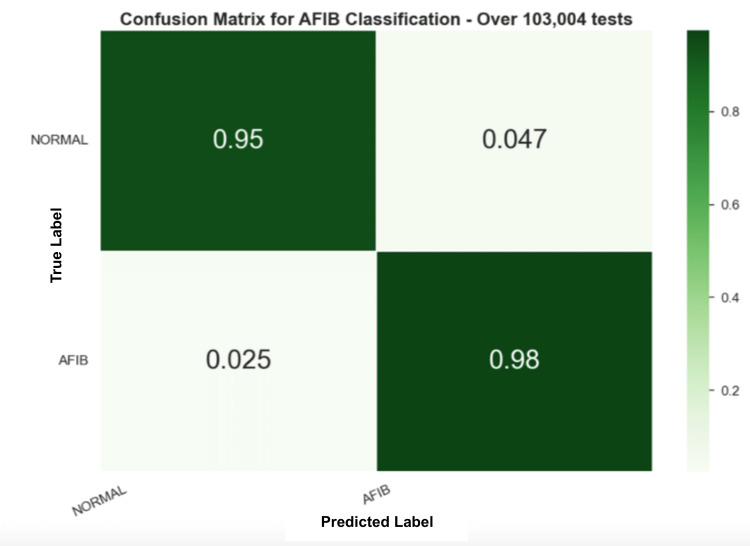
Confusion matrix for the fourth experiment with the RF model Random Forest (RF)

In Figure 9, the confusion matrix for the ML method was applied to the AFDB testing set. However, this experiment evaluates each HRV entry individually, which means that the model is predicting a label for each frame. Therefore, it is also possible to put all the frames’ predictions together and choose the final prediction of the sample based on a voting system. Figure 10 shows a random forest model with the confusion matrix for AFIB classification.

In the first experiment of the ML method, the remaining 40% of the AFDB data, which was not used to train the models, was used as the test set (Table 1).

**Table 1:** Statistical performance of each model Arial Fibrillation Database (AFDB), Normal Sinus Rhythm Database (NSRD), Atrial Fibrillation Contraction (AFC)

Dataset	Acc. %	Prec. %	Recall %	F1-score %
AFDB	90.82	90.98	91.09	90.81
AFC	87.32	73.59	86.28	77.49
NSRDB	92.3	-	-	-

As can be seen in Table 2, the RF model achieved the best performance with 96.03% accuracy, 95.08% precision, 96.38% recall, and an F1-score of 95.67%.

**Table 2:** Statistical results for all three models in the ML method with the AFDB testing set Machine Language (ML), Atrial Fibrillation Database (AFDB)

Model	Acc. %	Prec. %	Recall %	F1-score %
RF	96.06	95.08	96.38	95.67
SVM	95.29	92.73	93.84	93.27
KNN	90.3	89.21	91.53	89.9

**Figure 11:**
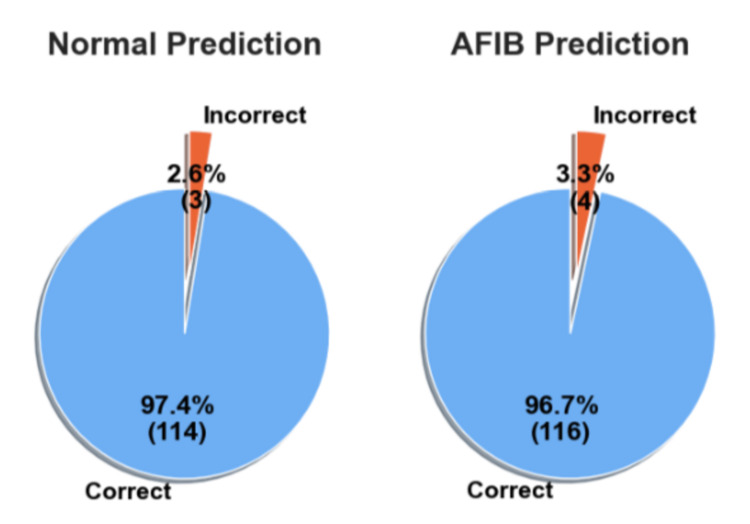
Correct and Incorrect predictions of normal and atrial fibrillation (AFIB) samples on the atrial fibrillation database (AFDB) testing set Atrial Fibrillation (AFIB), Atrial Fibrillation Database (AFDB)

In the second experiment, the models trained on the AFDB dataset were tested on all the data from the AFC dataset. Table 3 shows the statistical performance of each model. In this experiment, the SVM model achieved the best performance with 94.31% accuracy, 83.24% precision, 86.30% recall, and an F1-score of 84.68%. However, even though the SVM model achieved slightly better performance, this work chose to keep the analysis with the RF model due to the preference for ensemble methods. Figure 11 shows correct and incorrect predictions for normal and AFIB prediction.

**Table 3:** Statistical results for all three models in the ML method with the AFC dataset Machine Language (ML), Atrial Fibrillation Classification (AFC)

Model	Acc. %	Prec. %	Recall %	F1-score %
RF	93.46	80.62	86.56	83.23
SVM	94.31	83.24	86.3	84.68
KNN	92.08	77.32	85.08	80.5

**Figure 12:**
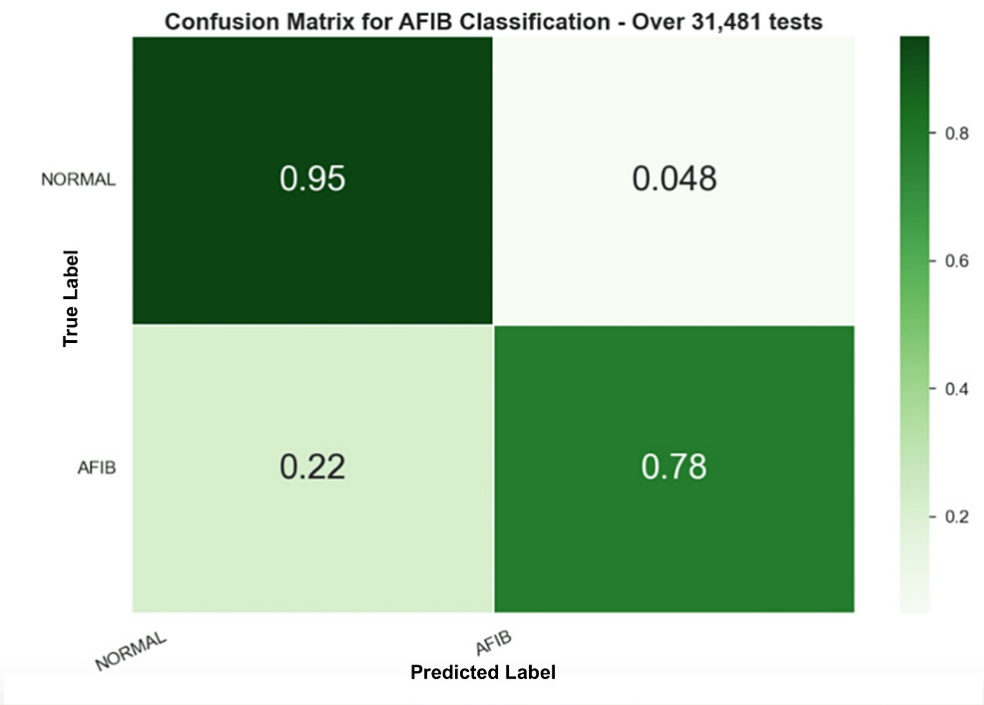
Shows the confusion matrix for the fifth experiment with the Random forest (RF) model Random forest (RF)

Similar to the previous experiment, it is possible to evaluate the results by choosing the final prediction of the sample using a voting system within the frames’ predictions. Figure 12 shows the confusion matrix with the random forest model for the fifth experiment.

The last experiment seeks to evaluate the false positive rate. One of the challenges when using ML models is to make them robust enough for false positives. As stated previously, the NSRDB only has normal samples; thus, the model was able to correctly predict 41,703 out of 44,399 HRV frames, resulting in 93.93% accuracy.

When grouping all the frames from respective samples, the model was able to predict correctly as normal in 13 out of 14 samples, resulting in 92.86% accuracy. Moreover, Table 4 shows the summary of the statistical results of the ML method when applied to three different datasets with HRV frames.

**Table 4:** Statistical results for the ML method in all three datasets Machine Learning (ML)

Dataset	Acc. %	Prec. %	Recall %	F1-score %
AFDB	96.06	95.08	96.38	95.67
AFC	93.46	80.62	86.56	83.23
NSRDB	93.93	-	-	-

Once the ECG tests were conducted, we investigated whether our methods could be applied to PPG signals or not. Even though ECG-based solutions can bring a massive improvement to healthcare areas in detecting AFIB, those still rely mostly on electrodes to properly extract the signal. Thus, a PPG-based solution could be embedded in small devices and be used at home on a daily basis. In our study, we propose an evaluation of our methods applied to two different datasets, a subset of the MIMIC-III dataset and the UMMC Simband dataset.

At this point, based on previous results and analysis, we chose the RF ML method trained using the AFDB dataset as the final solution. The results presented in the next two sessions will describe the performance of the trained ML solution using only PPG signals as the test set.

In this solution, two signal quality layers were added in an attempt to prevent poor signals from being predicted, leading to wrong predictions. The first layer is based on the previous method, which uses the IBI signal to detect invalid peaks. Hence, the signal must have at least 75% of the valid peaks to pass this test. Moreover, the signal-noise ratio (SNR) was used as an additional layer to prevent making predictions on poor-quality signals. In this work, the signal must have a mean SNR greater than 5 dB to be considered a valid signal.

**Figure 13:**
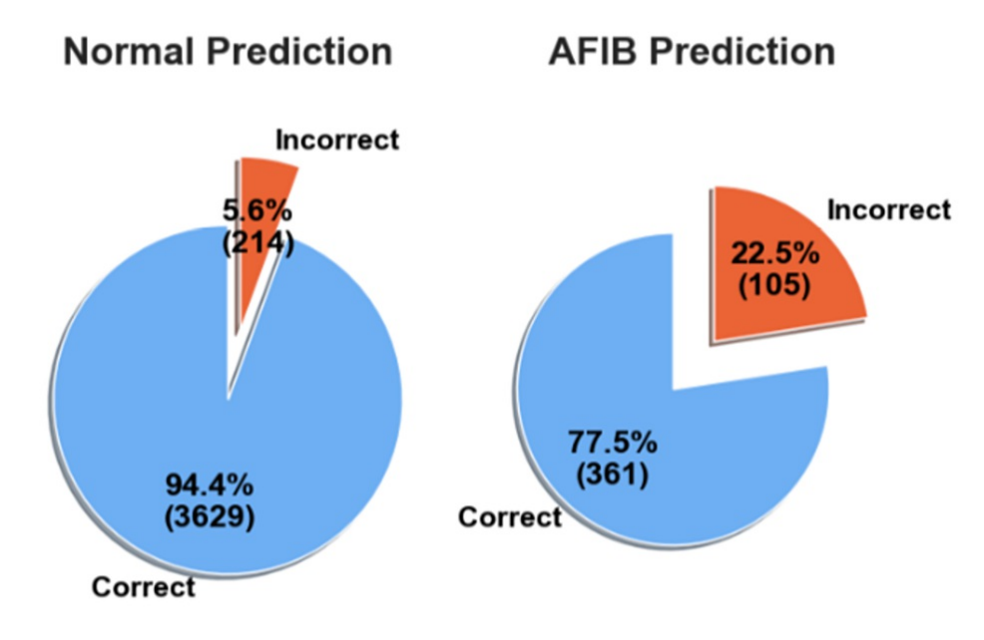
Correct and incorrect predictions of normal and atrial fibrillation (AFIB) samples on the atrial fibrillation classification (AFC) dataset Atrial Fibrillation (AFIB), Atrial Fibrillation Classification (AFC)

In the first PPG experiment, we segmented the PPG signals from the MIMIC-III dataset into 30-s samples. Then, the two-step signal quality check was used to remove bad-quality signal segments. HRV features were extracted from the remaining signal segments that passed the two-step quality check. Figure 8 shows the dataset distribution. The features extracted were used as inputs into the trained model. The results were collected and presented in Table 5 with statistical metrics, as well as the confusion matrix in Figure 13.

**Table 5:** Statistical results for random forest machine language (RF ML) model trained using the atrial fibrillation database (AFDB) dataset and tested on microwave monolithic integrated circuit (MIMIC)-III dataset

Model	Acc. %	Prec. %	Recall %	F1-score %
RF	97.32	96.68	94.37	95.47

**Figure 14:**
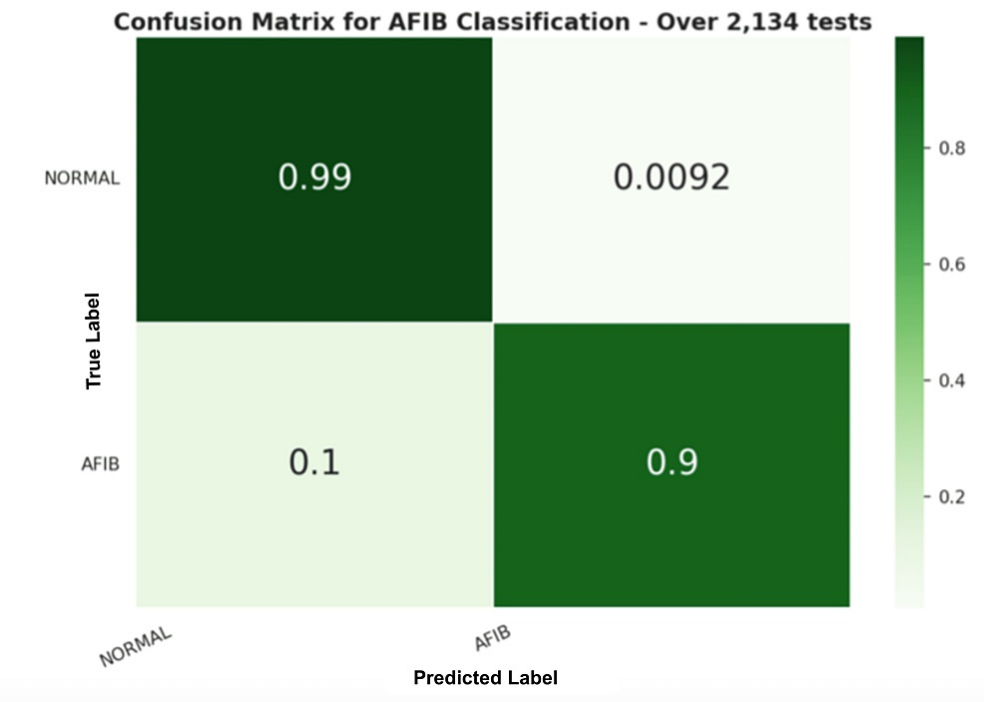
Confusion matrix for the ML method applied to the MIMIC-III PPG dataset Machine Language (ML), Microwave Monolithic Integrated Circuit (MIMIC), Photoplethysmography (PPG)

**Figure 15:**
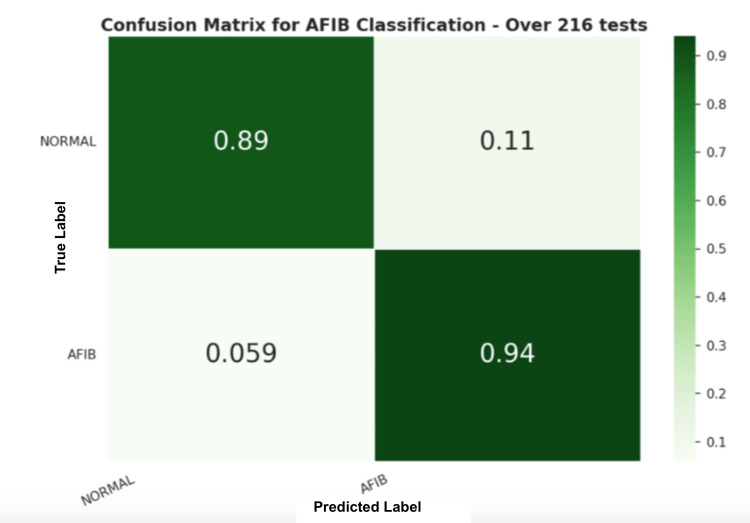
Confusion matrix for the ML method applied to the UMMC PPG dataset Machine Learning (ML), University of Mississippi Medical Center (UMMC), Photoplethysmography (PPG)

Secondly, the UMMC dataset was used to perform another round of tests on the trained model. Similarly, 30-s window signals were extracted, processed, and evaluated using the two-step signal quality check. Figure 8 shows the dataset distribution. Table 6 and Figure 14 show the results. Figure 15 shows the confusion matrix for the machine language method.

**Table 6:** Statistical results for the RF ML model trained using the AFDB dataset and tested on the UMMC dataset Random Forest (RF), Machine Language (ML), Atrial Fibrillation (AFDB) Dataset, University of Mississippi Medical Center (UMMC) Dataset

Model	Acc. %	Prec. %	Recall %	F1-score %
RF	89.81	80.15	91.56	84.03

Ultimately, Table 7 summarizes the RF model’s results across all five datasets.

**Table 7:** Statistical results for the ML method in all five datasets Machine Learning (ML)

Dataset	Acc. %	Prec. %	Recall %	F1-score %
AFDB	96.62	96.62	96.62	96.62
AFC	92.64	80.08	86.06	82.68
NSRDB	92.86	-	-	-
MIMIC-III	97.32	96.68	94.37	95.47
UMMC	89.81	80.15	91.56	84.03

## Discussion

The proposed methods show robust and reliable results across three different datasets. The DSP method does not need a training process; however, it does need an optimal threshold to be chosen to correctly identify and avoid noisy parts of a signal. The optimal threshold was set based on experiments to determine the underlying baseline. In this work, the chosen threshold is 50 ms.

In the first experiment, as can be seen in Figure 6, the DSP method was able to achieve an F1-score of 90.81% with 95% of predictions correctly identified as AFIB and 13% as false positives, which shows a high rate of reliability on the model. Furthermore, the DSP method underwent testing on an additional dataset, as depicted in Figure 7. Despite a reduction in performance compared to the prior findings, the outcomes remain satisfactory, accurately identifying 85% of predictions as AFIB. This implies that, in 15% of cases, the model did not detect an AFIB event. However, the false-positive rate remained with 12% of the normal predictions being incorrectly classified as AFIB.

Lastly, in the NSRDB dataset, which generally exhibits good quality signals with normal sinus rhythm data, the model was able to achieve 92.30% accuracy (12 correct predictions out of 13) and represents that the method is robust in detecting normal samples.

In the ML method, the datasets were highly augmented using HRV features extracted from individual frames, as can be seen in Figure 8. Following the extraction stage, the AFDB dataset had about 230,000 samples with almost two times more normal samples than AFIB. For the other datasets, we ended up with 31,000 samples from the AFC dataset and 116,000 from the NSRDB dataset.

Unlike the DSP method, the ML method does require a training process, and 60% of the data from the AFDB dataset was used to train the model. However, 60% were from individual samples to ensure that multiple samples from each individual do not end up in both the training and test sets. Three different ML models were compared in this work and were evaluated on the remaining 40% of the data. Results described in experiment 4 show that the random forest model performed the best, with an F1-score of 95.67%. Moreover, Figure 9 shows that the RF model achieved higher performance on the test set with 98% of the samples correctly identifying AFIB predictions.

Furthermore, these results show the ability of the model to predict frame-by-frame, or in other words, for every 15-s window. This behavior is interesting given its applicability to real-time applications. Furthermore, the DSP method underwent testing on an additional dataset, as depicted in Figure 7. Despite a reduction in performance compared to the prior findings, the outcomes remain satisfactory, accurately identifying 85% of predictions as AFIB. This implies that, in 15% of cases, the model did not detect an AFIB event. Thus, the frames’ predictions were grouped by ID, and a voting system was used to decide the final outcome. Across 114 samples (97.4%), the RF model correctly identified the non-AFIB diagnosis with only three errors, while 116 AFIB samples were correctly identified (96.7%) with only four errors. These results show that the model had success in detecting AFIB reliably across the test set.

However, in order to corroborate the results, the trained models were tested on a completely unseen dataset and without additional training. In the AFC dataset, the SVM model performed slightly better, as shown in Table 3; however, this work chose to continue the analysis with the RF model due to the performance in further tests as well as the characteristics of an ensemble method. The RF model was able to achieve an F1-score of 83%, which represents, as described in Figure 11, 95% of the normal and 78% of the AFIB predictions correctly identified. Given the decrease in performance, we decided to apply a similar vote method, as we did in the previous test. As can be seen in Figure 12, the performance was almost the same as the frame predictions, for normal and AFIB predictions, where there were 3,629 correctly identified normal samples with 214 errors and 361 correct AFIB predictions with 105 errors.

Finally, in the NSRDB dataset, the RF model was able to achieve almost 94% accuracy in predicting normal ECG signals in the frame-to-frame experiment. Moreover, when predicting the final outcome the model was able to predict correctly 13 out of 14 samples (92.86%).

All the results from the three datasets were combined in Table 4, showing that the RF model was able to achieve considerable results in detecting AFIB events as well as normal samples, with an F1-score greater than 80% for all the datasets.

Moreover, experiments 7 and 8 describe the trained RF model tested on two other datasets, which consisted of PPG signals only. Using a subset of the MIMIC-III dataset as the unseen test set, the RF model was able to achieve an F1-score of 95%, where 99% of the normal and 90% of AFIB predictions were correctly classified, as described in Figure 13.

Similarly, using the UMMC dataset the trained RF model was able to achieve an F1-score of 84%, with 89% of normal and 94% of AFIB predictions correctly identified. These results show that the ECG-based trained model was able to correctly identify AFIB events in PPG signals; moreover, this has shown robustness against false positives.

Ultimately, this work proposes a comparison with other authors who, in their work, tried to achieve the same goal, AFIB detection using non-invasive real-time techniques. Table 8 compares three other authors, each one with a different approach; however, all of them use a single dataset, AFDB.

**Table 8:** Performance of the classification methods for atrial fibrillation detection Digital Signal Processing (DSP), Radio-Frequency Machine Learning (RFML)

Authors	Accuracy %	Recall %
This work - DSP	90.82	91.09
This work - RF ML	96.06	96.38
Ross-Howe et al. [7]	93.16	98.33
Bruun et al. [9]	93.33	87.97
Hu et al. [10]	96.3	88.7

Through the comparison shown in Table 8, as can be seen in the recall metric, this work, using both DSP and the ML model, had slightly better performance than two authors [9] and [10], while the approach proposed by Ross-Howe et al. [7] achieved the best performance with 98.33% recall.

## Conclusions

In this paper, we proposed two different methods for AFIB detection using short-term ECG signals. The first method used only signal processing techniques to identify abrupt changes in the first derivative of the IBI signal. Using the ratio of abrupt changes with the number of peaks in a given frame, the DSP method classifies the condition using an optimal threshold. In contrast with the DSP method, ML models were used. Seven HRV features were extracted from a 15-s ECG window and were used as input for different ML models. Several experiments were conducted using three different ECG datasets that are publicly available. In the DSP method, all the data were used for testing, since there is no training process, while the ML models were trained using 60% of the individuals available in the AFDB dataset. Results have shown that the DSP method was able to achieve 90.82%, 87.32%, and 92.30% accuracy, respectively, in the AFDB, AFC, and NSRDB datasets. Similarly, the RF ML model was able to achieve 96.62%, 92.94%, and 92.86% accuracy, respectively. The ECG-based trained RF ML model was chosen as the final solution and tested on two PPG datasets, without additional training. Reported results show the F1-scores of 95.47% and 84% for the MIMIC-III and UMMC datasets, respectively.

Ultimately, this work proposed a comparison with other authors that used the AFDB dataset to predict AFIB. This work showed slightly better performance when compared with the two of them. Even though this work has certain limitations against poor signal quality and noise influence, the benefits of identifying AFIB events in short-term ECG and PPG signals are a major breakthrough in healthcare research and large-scale monitoring. One of the primary distinctions of our research lies in the vast and diverse datasets utilized. With 269,842 signal segments spanning five databases, our study offers one of the most comprehensive analyses in this area. This diverse data foundation allows us to provide insights that might be more generalizable than those derived from studies based on smaller or less varied datasets. Moreover, while ML models have been used for AFIB detection using ECG or PPG signals individually, our study dives deeper into the hypothesis that models trained on ECG segments can be repurposed to predict AFIB from PPG segments. This exploration opens potential avenues for more flexible AFIB detection tools that can function across data modalities. Lastly, our research does not solely focus on presenting a new method but also compares two distinct approaches to detect AFIB from short-term ECG and PPG signals. This comparative analysis provides readers with a broader perspective on the efficiency and efficacy of different methods.
